# Mr. Alexander Nasmyth

**Published:** 1849-01

**Authors:** 


*Obituary.*—The death of Mr. ALEXANDER NASMYTH, has been communicated to us by Dr. Robert Nasmyth, son of Dr. Robert Nasmyth of Edinburg, and brother of the deceased. He died, as we learned from the gentleman above mentioned, in August, 1848. By this sad event, science has lost one of her ablest and most zealous votaries, and the dental profession one of its brightest ornaments. It is known, perhaps to many, that for many years previously to his death, Dr. N., was engaged in the preparation of a work on the development, structure and diseases of the teeth, and the writer of this notice received a letter from him in 1846, informing him that it was then nearly ready for the press, and would be published in a short time. For the preparation of a treatise of this sort no one was more eminently qualified; and it is sincerely to be hoped, that the profession will not be deprived of the results of the valuable labors of so scientific and distinguished a man. The historical introduction to this treatise, a work characterized by great research and ability, has been extensively read and much admired, and which, together with three memoirs on the structure of the teeth and epithelium, will perpetuate his name to the latest period of time. Still we cannot but cherish the hope, that the other work will soon be published. It would meet with a rapid and extensive sale on this side of the Atlantic.—*Balt. Ed.*

